# Sappanone A Alleviates the Severity of Carbon Tetrachloride-Induced Liver Fibrosis in Mice

**DOI:** 10.3390/antiox12091718

**Published:** 2023-09-04

**Authors:** Jing Qi, Lanqian Li, Xueqing Yan, Wenxi Hua, Zixiong Zhou

**Affiliations:** 1 Department of Biochemistry and Molecular Biology, The School of Basic Medical Sciences, Fujian Medical University, No. 1, Xuefu North Road, University Town, Fuzhou 350122, China; yanxueqing@fjmu.edu.cn; 2 Department of Pathology and Institute of Oncology, The School of Basic Medical Sciences, Fujian Medical University, Fuzhou 350122, China; llq1998@fjmu.edu.cn (L.L.); jchwx@fjmu.edu.cn (W.H.); 3 Diagnostic Pathology Center, Fujian Medical University, Fuzhou 350122, China

**Keywords:** liver fibrosis, sappanone A, oxidative stress, inflammation, M2 polarization

## Abstract

Liver fibrosis is a major challenge to global health because of its various complications, including cirrhosis and hepatocarcinoma, while no effective treatment is available for it. Sappanone A (SA) is a homoisoflavonoid extracted from the heartwood of *Caesalpinia sappan* Linn. with anti-inflammatory and antioxidant properties. However, the effects of SA on hepatic fibrosis remain unknown. This study aimed to investigate the protective effects of SA on carbon tetrachloride (CCl_4_)-induced liver fibrosis in mice. To establish a liver fibrosis model, mice were treated intraperitoneally (i.p.) with CCl_4_ for 4 weeks. SA (25, 50, and 100 mg/kg body weight) was i.p. injected every other day during the same period. Our data indicated that SA decreased liver injury, fibrotic responses, and inflammation due to CCl_4_ exposure. Consistently, SA reduced oxidative stress and its-mediated hepatocyte death in fibrotic livers. Of note, SA could not directly affect the activation of hepatic stellate cells. Mechanistically, SA treatment lessened oxidative stress-triggered cell death in hepatocytes after CCl_4_ exposure. SA down-regulated the expression of M1 macrophage polarization markers (CD86 and iNOS) and up-regulated the expression of M2 macrophage polarization markers (CD163, IL-10, and Arg1) in livers and macrophages. Meanwhile, SA induced the activation of peroxisome proliferator-activated receptor gamma (PPARγ). However, decreased inflammatory responses and the trend of M2 macrophage polarization provided by SA were substantially abolished by SR202 (a PPARγ inhibitor) treatment in macrophages. Additionally, SA treatment promoted fibrosis regression. Taken together, our findings revealed that treatment with SA alleviated CCl_4_-induced fibrotic liver in mice through suppression of oxidative stress-mediated hepatocyte death and promotion of M2 macrophage polarization via PPARγ. Thus, SA might pave the way for a new hepatoprotective agent to treat liver fibrosis.

## 1. Introduction

Liver fibrosis is the outcome of dysregulated wound-healing responses due to a variety of chronic liver diseases, such as viral hepatitis, nonalcoholic steatohepatitis (NASH), alcohol-induced liver injury, cholestasis, drug-induced liver injury, and autoimmune hepatitis [1]. One of the main features of liver fibrosis is the accumulation of an extracellular matrix (ECM), such as collagen, in the subendothelial space. This sustained hepatic injury can be transformed into liver dysfunction and cirrhosis (the late and irreversible stage of hepatic fibrosis). Furthermore, liver fibrosis has been recognized as a pre-cancerous circumstance in that 60–80% of hepatocellular carcinoma (HCC) patients might have arisen from cirrhosis [2]. Moreover, liver fibrotic reactions are deeply united with the onset of many kinds of life-threatening complications, including varix, ascites, hepatic encephalopathy, hepatopulmonary hypertension, and renal failure [3]. Despite tremendous advances in anti-fibrotic therapy and fibrosis reversal in experimental research that have been developed within the past decades, liver transplantation is the main curative approach for patients with advanced hepatic fibrosis or cirrhosis. Hence, it is of great clinical significance to explore effective treatments based on understanding the pathophysiologic mechanism of hepatic fibrosis at mild and moderate phases due to its reversibility.

Carbon tetrachloride (CCl_4_) is commonly used to establish experimental animal liver fibrosis models because this animal model is highly accessible, reproducible, and appropriately reflects the mechanisms of human liver fibrosis/cirrhosis. CCl_4_ causes hepatocyte damage, liver inflammation, and liver fibrosis after 4 weeks of the challenge, and over 8 weeks, it causes liver cirrhosis. Furthermore, the CCl_4_-evoked murine liver fibrosis model is combined with the scarcity of bone marrow hemopoiesis indicated by damaged maturation of hemopoietic cells and a significant reduction in erythroid precursors and granulocyte-macrophage. Additionally, the CCl_4_ challenge induces harsh anemia, neutrophilia, lymphocytopenia, leucopenia, eosinophilia, and haemoglobinaemia, along with changes in plasma globulin and albumin [4]. Therefore, it is important to evaluate the cytotoxicity of potential anti-fibrotic drugs on hematopoietic indices in CCl_4_-challenged mice because the number of blood cells is indispensable for the homeostasis of immune systems, which contributes to identifying the end-point toxicity.

The pathophysiologic process that characterizes hepatic fibrotic responses is complicated, involving hepatocyte necroptosis, Kupffer cell (KC) activation and recruitment, and hepatic stellate cell (HSC) activation. During the early stages, long-term hepatic insults induce hepatocyte death, which triggers KC activation and stimulates the secretion of pro-inflammatory cytokines and pro-fibrogenic cytokines, such as transforming growth factor beta (TGF-β), which subsequently activates HSC in a paracrine manner. Furthermore, the apoptotic bodies released from damaged hepatocytes can directly activate HSC in myofibroblasts [5]. These cellular communications lead to the accumulation of extracellular matrix components, including collagen, thus resulting in hepatic fibrosis. In addition, hepatic inflammation, generating reactive oxygen species (ROS), and ROS-triggered oxidative stress contribute to the pathogenesis of the initiation and progression of liver fibrosis.

Peroxisome proliferator-activated receptor-γ (PPARγ) is known to be a ligand-dependent transcriptional regulator, which belongs to a superfamily of hormone nuclear receptors. It is well accepted that PPARγ has a variety of biological functions, including regulating lipid metabolism, HSC activation, macrophage polarization, and inflammatory responses [6]. A previous study reported that PPARγ sumoylation mediated by ligand binding reduces inflammation via transrepression of nuclear factor kappa B (NF-κB) target genes in macrophages [7]. Furthermore, PPARγ phosphorylation by c-Src represses macrophage activation and its related inflammatory reactions in adipose tissues [8]. Moreover, our previous studies have demonstrated that PPARγ activation promotes M2 polarization of hepatic macrophages, thus decreasing NASH-related inflammation, lipid accumulation, and liver fibrosis in mice [9]. Therefore, PPARγ has become a candidate target for treating inflammatory-related diseases, including colitis, atherosclerosis, and asthma [10,11,12]. Following other inflammatory diseases, inhibiting PPARγ by interleukin 2 or interferon-gamma in intrahepatic biliary epithelium exacerbated the progression of chronic cholangitis in a primary biliary cirrhotic murine model [13]. Similarly, in the CCl_4_-induced liver fibrosis model, PPARγ deletion in KC reveals pro-inflammatory and pro-fibrotic responses, while such impacts are not found in hepatocyte-specific PPARγ knock-out mice [14]. Consistently, adenovirus-mediated overexpression of PPARγ or treatment with rosiglitazone, a PPARγ agonist, alleviates inflammatory responses and liver fibrosis in a NASH murine model [15,16].

Natural products are a great source of candidate drugs to treat liver diseases. Various active compounds extracted from natural products exert inhibitory effects on KC or HSC activation characterized by decreased ECM deposition, thus having potential values to treat or prevent liver fibrosis [17]. Among many other phytochemicals, silymarin, curcumin, and resveratrol are the most extensively studied natural products with potential anti-fibrotic activity [18]. However, these therapeutic candidates for liver fibrosis have hit a bottleneck in clinical trials. Low water solubility and limited oral bioavailability due to poor enteral absorption (23–47%) and high first-pass metabolism in the liver hamper the use of silymarin in clinical trials [19]. Similar to silymarin, curcumin is characterized by low oral bioavailability [20]. In a randomized and double-blinded clinical trial in nonalcoholic fatty liver disease (NAFLD) patients, treating resveratrol orally for 12 weeks compared to placebo showed significantly decreased hepatic steatosis and inflammation, but did not affect liver fibrosis [20]. Therefore, more therapeutic candidates for treating liver fibrosis need to be investigated. Sappanone A (SA) is an active component extracted from the dry heartwood of *Caesalpinia sappan* Linn. SA has been reported to have various pharmacological effects, including antioxidative effects, anti-inflammatory effects, and anti-apoptotic effects [21,22]. A previous study has demonstrated that SA treatment decreased Lipopolysaccharide (LPS)-induced inflammatory responses by inhibiting NF-κB signaling in macrophages [23]. Similarly, recent studies reported that SA has anti-inflammatory effects in multiple chronic inflammatory diseases [24,25]. Based on the association between chronic inflammatory diseases and liver fibrosis, we hypothesized that SA might ameliorate the severity of liver fibrosis.

## 2. Materials and Methods

### 2.1. Plant Material

SA (CAS#102067-84-5) was purchased (Yuanye Bio-Technology Co., Ltd., Shanghai, China). High-performance liquid chromatography analysis was used to analyze SA quantitatively, and data showed a purity of more than 98%.

### 2.2. Animals

Seven-week-old male C57BL/6N mice (Cyagen Biosciences Inc., Guangzhou, China) were housed under a specific pathogen-free condition (12 h (h) light/dark cycle, 50 ± 5% relative humidity, between 22 and 26 °C) with free access to safe drinking water and food. All animal protocols are approved by the Laboratory Animal Research Center of Fujian Medical University (ethical code: FJMU IACUC 2021-J-0556).

To induce hepatotoxin-mediated liver fibrosis, mice were treated intraperitoneally (i.p., 2 mL/kg body weight) with CCl_4_ (2:5 *v*/*v* in corn oil, Sigma-Aldrich, St. Louis, MO, USA) or the equal volume of corn oil (Sigma-Aldrich) three times per week, for consecutive 4 weeks.

As shown in Table 1, a total of 72 mice were randomly divided into 8 groups. To estimate the impacts of SA on CCl_4_-induced liver fibrosis, mice in each group were treated with SA (25, 50, and 100 mg/kg body weight, i.p.) or an equal volume of vehicle every two days during the experiment.

As previously mentioned, with a slight modification (the resolution) [26], mice discontinued the toxin within the first 2 weeks after the CCl_4_ injection to establish the model of fibrosis resolution. During discontinuation of the toxin, mice were administrated with SA (100 mg/kg body weight, i.p.) or an equal volume of vehicle every two days (*n* = 6 mice/group). Animals fasted for 8 h before the necropsy. Tissues were collected with standard necropsy techniques for further analysis.

### 2.3. Biochemical Assays

To measure the severity of liver injury induced by hepatotoxin, alanine aminotransferase (ALT) and aspartate aminotransferase (AST) levels in serum were determined with assay kits purchased from Nanjing Bioengineering Institute (Nanjing, China).

Hepatic hydroxyproline contents, the important diagnostic indicator of the severity of fibrosis, were quantified with the hydroxyproline assay kit purchased from Nanjing Bioengineering Institute (Nanjing, China) according to the manufacturer’s instructions. An Emax Precision Microplate Reader (ThermoFisher Scientific Oy Ratastie, Vantaa, Finland) was used to detect the absorbance at 560 nm. Malondialdehyde (MDA), glutathione (GSH), and superoxide dismutase (SOD) levels in mice liver were measured by using the MDA assay Kit (S0131S, Beyotime, Shanghai, China), the GSH and GSSG quantification kit (S0053, Dojindo, Kumamoto, Japan), and the SOD kit (S0101S, Beyotime, Shanghai, China). MDA, a common marker of lipid peroxidation, can condense with thiobarbituric acid (TBA) to form red MDA-TBA adduct in acidic environments at 95 °C; detailed procedures were reported by Qinglei Xu et al. [27]. For GSH assay, as we described before [28], the detection principle is that GSH can react with the substrate DTNB to produce stable yellow TNB and GSSG. Principle of SOD detection kit is as follows: superoxide anion, produced by xanthine and xanthine oxidase reaction system, can reduce nitrogen blue tetrazole to produce blue methylate, which is absorbed in 560 nm; SOD can clear O_2_^−^, thus inhibiting the formation of formazan [29].

### 2.4. Staining of Liver Sections

As previously described [30], liver tissues were fixed in 10% neutral buffered formalin for 48 h at room temperature, processed and embedded in paraffin, and then manually sliced to obtain 4 μm-thick paraffin sections. Hematoxylin and eosin (H&E) staining was used for morphological studies.

The intensity of hepatic necrosis was scored according to the extent of the lesion. Grade 0, no pathological change; Grade 1, rare necrotic lesions with the existence of degenerated hepatocytes; Grade 2, scattered necrotic hepatocytes; Grade 3, confluent necrosis; and Grade 4, severe damage.

Masson’s trichrome kit (G1340, Beijing Solarbio Science & Technology Co., Ltd., Beijing, China) and Sirius Red staining kit (BP-DL029, Nanjing SenBeiJia Biological Technology Co., Ltd., Nanjing, China) were used to evaluate the severity of liver fibrosis. The images of the liver section were produced using a light microscope (BX-51, Olympus Corp., Tokyo, Japan). The analysis and evaluation of all images were processed using Image J software (V1.8.0.112).

To explore cell death, terminal deoxynucleotidyl transferase-mediated dUTP nick end labeling (TUNEL) staining (Biotin TUNEL Assay Apoptosis Detection Kit, Life-iLab, Shanghai, China) was employed following the manufacturer’s protocol. The percentage of the TUNEL-positive cell in the entire field for 3 different visual fields was calculated using Image J software (V1.8.0.112).

F4/80, CD86, and CD163 positive cells were stained by using immunohistochemistry (IHC) to investigate macrophage infiltration in the livers of mice. The protocols were described in detail in our study [31].

### 2.5. Quantitative Real-Time Polymerase Chain Reaction (qRT-PCR)

FastPure^®^ Cell/Tissue Total RNA Isolation Kit (Vazyme, Nanjing, China) and Trizol reagent were used to extract total RNA from liver tissues or cells, followed by the measurement of RNA concentrations. The HRbio™ III 1st Strand cDNA Synthesis Kit (OneStep gDNA Removal) for qRT-PCR was used to convert RNA into cDNA (Fujian Herui Biotechnology Co., Ltd., Fuzhou, China). A PCR Master Mix was utilized to perform qRT-PCR and evaluate the gene expression levels (HRF0032, HRbio^TM^ qPCR SYBR Green Master Mix, Herui, Oklahoma City, OK, USA). The primer sequences employed in this study are listed in Table 2. Glyceraldehyde-3-phosphate dehydrogenase was applied to normalize the relative gene expression. The comparative CT method was used to calculate the relative gene expression.

### 2.6. Enzyme-Linked Immunosorbent Assay (ELISA)

ELISA kits were used to evaluate the levels of tumor necrosis factor-alpha (TNFα), interleukin 6 (IL-6), and interleukin 1 beta (IL-1β) in livers of mice (Invitrogen, Carlsbad, CA, USA) following the direction of the manufacturer, which measures inflammatory responses in livers of mice.

### 2.7. Cell Culture and Treatments

AML12 cells obtained from Procell Life Science&Technology Co., Ltd. (Wuhan, China) were seeded in a 12-well plate (5.0 × 10^5^ cells/well) and maintained in Dulbecco’s modified Eagle’s medium (DMEM; Hyclone, Logan, UT, USA) containing 100 IU/mL penicillin, 100 μg/mL streptomycin, and 10% fetal bovine serum (FBS), in a humidified incubator at 37 °C in 5% CO_2_.

RAW 264.7 cells obtained from Procell were seeded in a 12-well plate (1.0 × 10^6^ cells/well) and cultured in RPMI-1640 containing 100 IU/mL penicillin, 100 μg/mL streptomycin, and 10% FBS.

Human HSC line (LX-2) obtained from Procell was routinely cultured in a 12-well plate (1.0 × 10^6^ cells/well).

To examine the impacts of SA on hepatocytes, AML12 cells were incubated with 0.3% CCl_4_ with or without SA for 24 h. To investigate the roles of SA in HSCs, LX-2 cells were incubated with 10 ng/mL human recombinant TGF-β with or without SA for 24 h. To explore the effects of SA on inflammation, RAW 264.7 cells were incubated with the indicated concentrations of SA or vehicle for 24 h. Then, LPS (1 μg/mL) was used to induce inflammation in RAW 264.7 cells. SR202 was used to inhibit PPARγ. RAW 264.7 cells were incubated with 2 μM SR202 and/or 100 μM SA at 30 min and 1 h after LPS (1 μg/mL) treatment. The samples, including cells and supernatants, were collected at 24 h after treatments.

### 2.8. Lactate Dehydrogenase (LDH) Assay

The LDH assay kit was purchased from Nanjing Bioengineering Institute (Nanjing, China) to detect LDH released in the cell supernatant. An Emax Precision Microplate Reader was utilized to measure the absorbance at a wavelength of 490 nm.

### 2.9. Cell Viability Assay

Cells were seeded into a 96-well plate and routinely incubated. Then, cells were treated with indicated concentrations of SA with or without CCl_4_ for 24 h. The cell viability was determined using an MTT assay (M1020, Solarbio Science & Technology Co., Ltd., Beijing, China). The detection principle is that the succinate dehydrogenase in the mitochondria of living cells can reduce the exogenous MTT to the water-insoluble blue–violet crystal formazan and deposit it in the cells, while the dead cells have no such function. Dimethyl sulfoxide (DMSO) can dissolve the formazan in cells, and the light absorption value can indirectly reflect the number of living cells. The absorbance at a wavelength of 570 nm was quantified using the Emax Precision Microplate Reader.

### 2.10. Statistical Analysis

Statistical significance was determined using GraphPad Prism software (version 9.3); all results are displayed as the mean ± standard deviation (SD). By applying a one-way analysis of variance (ANOVA) and using Tukey’s post hoc testing, the significance of differences between multiple groups was compared. Different letters were used to show significant differences between groups. By applying a two-tailed Student’s *t*-test, the significance of differences between the two groups was compared. *p* values of <0.05 were regarded to be statistically significant.

## 3. Results

### 3.1. Treatment with SA Alleviates Chronic Liver Injury in Mice

**Figure 1:**
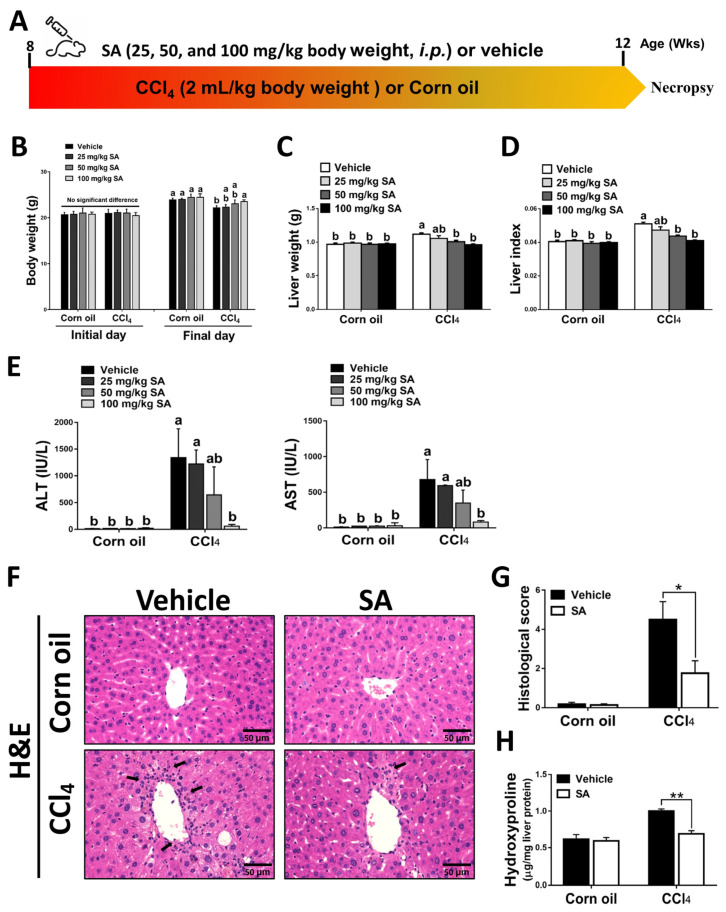
Sappanone A (SA) treatment ameliorates the severity of CCl_4_-induced liver injury in mice. (**A**) Mice were administered with CCl_4_ (2:5 *v*/*v* in corn oil, 2 mL/kg, i.p.) or an equal volume of corn oil three times a week for 4 weeks. Meanwhile, mice in each group were treated with SA (25, 50, and 100 mg/kg body weight, i.p.) or an equal volume of vehicle every other day. (**B**–**D**) The body weight and liver weight were monitored, and the liver index was calculated. (**E**) Serum ALT and AST levels were evaluated. (**F**) H&E staining. (**G**) The necrotic lesions in H&E-stained liver sections were scored. (**H**) Hepatic hydroxyproline levels were quantified. Data in each group are displayed as means ± SD. (**B**–**E**) Differences among multiple groups were analyzed with ANOVA followed by Tukey’s post hoc analysis. If there is at least one identical letter in the symbols of compared groups, there is no statistically significant difference between these groups. If there is no common letter in the compared groups, then the means between these groups are statistically different. (**G**,**H**) Differences between the two groups were compared by two-tailed Student’s *t*-test, * *p* < 0.05 and ** *p* < 0.01. Corn oil-treated groups (*n* = 8); CCl_4_-treated group (*n* = 10). Original magnification: ×400. The arrows in the picture show areas of inflammation or necrosis.

Mice were administered with CCl_4_ (2:5 *v*/*v* in corn oil, 2 mL/kg, i.p.) or an equal volume of corn oil three times a week for 4 weeks (Figure 1A). The daily water and food intake in each group were monitored, but there was no significant difference among them (Supplementary Figure S1A,B). Also, the body weight and liver weight of each mouse were monitored (Figure 1B,C). The body weight of all experimental groups was increased on the final day compared to the initial day. Compared to the corn oil-treated mice, CCl_4_-injection decreased mouse body weights but increased liver weights, while SA treatment substantially reversed these effects. The ratio of liver weight/body weight (liver index) was dose-dependently restored in CCl_4_-treated mice after SA administration (Figure 1D). The possible effect of SA on liver function was assessed by collecting serum samples from mice and measuring serum levels of ALT and AST. We observed that SA at a high dose of 100 mg/kg significantly alleviated CCl_4_ injection-induced liver damage in mice (Figure 1E). Therefore, we chose 100 mg/kg SA for further studies and analysis. Similarly, reduced hepatic damage in the livers of SA-treated mice subjected to CCl_4_ was further confirmed by histopathologic examinations. In the CCl_4_-injected group, the structure of hepatic lobules was destroyed, the arrangement of the hepatic cell cord was chaotic, and the infiltration of inflammatory cells was increased. However, SA treatment markedly improved these effects (Figure 1F,G and Supplementary Figure S2). Additionally, the hepatic level of hydroxyproline, a collagen-related amino acid, was significantly decreased by SA treatment in fibrotic livers (Figure 1H). Collectively, we found that SA treatment could ameliorate CCl_4_-mediated chronic liver injury in mice.

### 3.2. SA Treatment Ameliorates CCl_4_-Induced Liver Fibrosis in Mice

**Figure 2:**
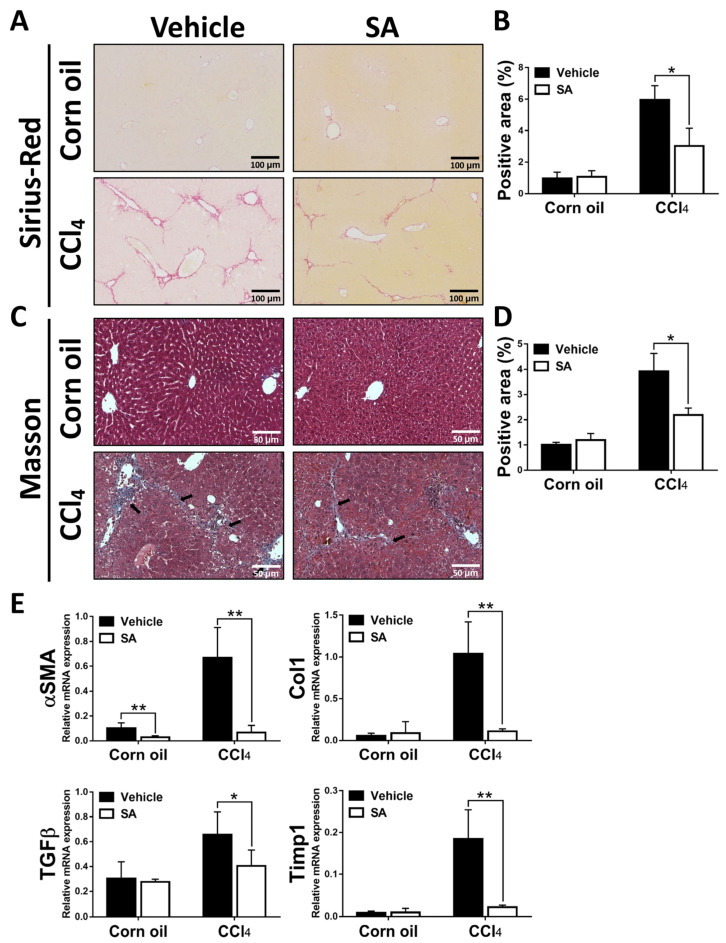
Sappanone A (SA) treatment alleviates the severity of hepatotoxin-induced liver fibrosis. Liver slides were stained with Sirius Red (**A**) or Masson (**C**) to explore hepatic fibrogenesis. Sirius Red positive area (**B**) and Masson positive area (**D**) were quantified. (**E**) The mRNA expression of αSMA, Col1, TGFβ, and Timp1 in liver tissues was measured by qRT-PCR. Data in each group are displayed as means ± SD. * *p* < 0.05 and ** *p* < 0.01. Corn oil-treated groups (*n* = 8); CCl_4_-treated group (*n* = 10). Original magnification: ×200 (Sirius Red); ×400 (Masson). The arrows in the picture indicate the collagen fibers.

Next, we determined whether SA has anti-fibrotic effects on CCl_4_-induced liver fibrosis. As shown in Figure 2A–D, Sirius Red and Masson staining of liver sections showed that a significant improvement in liver fibrosis was found in SA-treated fibrotic mice compared with the vehicle-treated mice, leading to thinner and less pronounced fibrous septa with frequent perforation. Consistently, treatment of SA reduced the expression of profibrogenic genes in the livers of fibrotic mice, including Asma, TGFβ, Col1, and Timp1 (Figure 2E). Overall, SA has anti-fibrotic properties in CCl_4_-induced liver fibrosis.

### 3.3. SA Protects Hepatocytes from Oxidative Stress Damage

**Figure 3:**
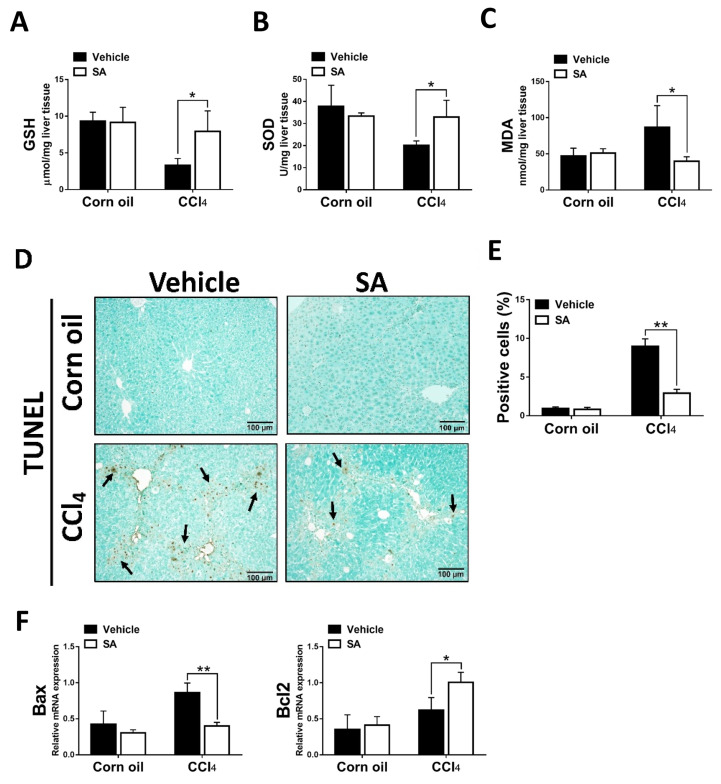
Treatment with sappanone A (SA) inhibits oxidative stress and apoptosis in damaged livers of mice. (**A**) GSH levels in the lives of mice were measured at 24 h. (**B**) The activity of SOD in the livers of mice was determined at 24 h. (**C**) MDA levels in the livers of mice at 24 h were evaluated to measure lipid peroxidation. (**D**) TUNEL assay was used to explore cell death in the livers of mice. (**E**) TUNEL-positive fields’ percentages were calculated. (**F**) The mRNA expression of Bax and Bcl2 in the livers of mice was investigated by qRT-PCR. Data in each group are shown as means ± SD. * *p* < 0.05 and ** *p* < 0.01. Corn oil-treated groups (*n* = 8); CCl_4_-treated group (*n* = 10). Original magnification: ×200. The arrows in the picture show the dead cells.

The clinical and experimental data indicated that oxidative stress mediated the progression of liver fibrosis because continuous oxidative insults induce hepatocyte death, subsequently resulting in hepatic inflammation and fibrosis [32]. In this study, the data showed that SA treatment notably up-regulated hepatic levels of GSH and SOD and, in parallel, reduced MDA contents in fibrotic livers (Figure 3A–C). Consistently, the TUNEL assay and analysis of TUNEL-positive cells (Figure 3D,E) revealed that SA treatment significantly lessened CCl_4_-induced hepatocyte cell death. Also, SA down-regulated the expression level of Bax while SA up-regulated the expression level of Bcl2 in injured livers (Figure 3F).

**Figure 4:**
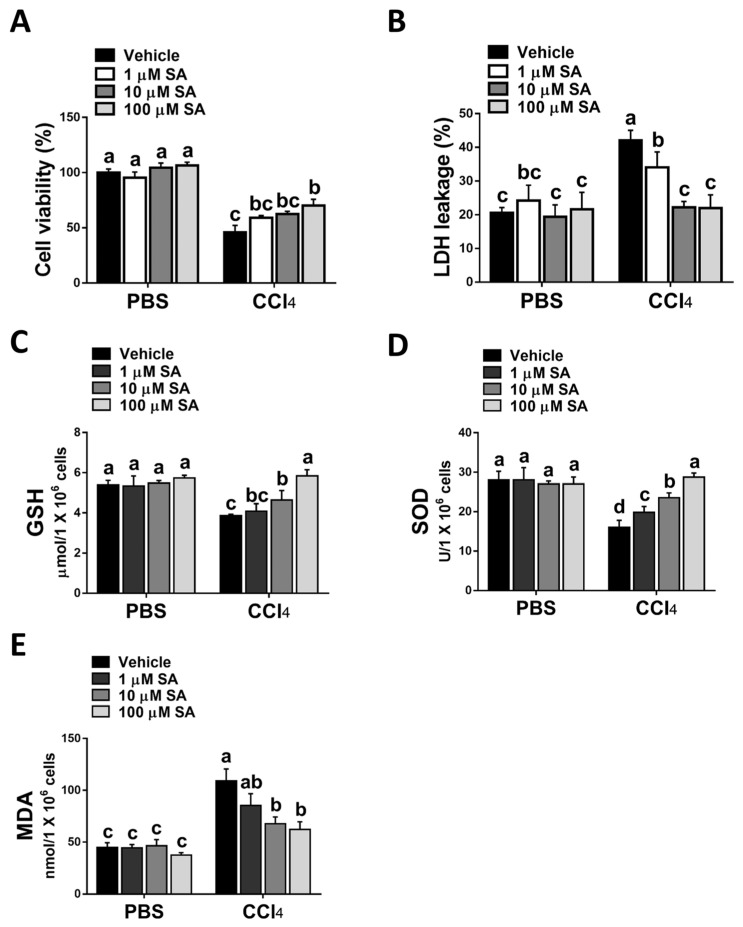
Sappanone A (SA) treatment reduces CCl_4_-induced hepatotoxicity in vitro. AML12 cells were stimulated with 0.3% CCl_4_. Cells were treated with SA (1, 10, and 100 μM) for 30 min after CCl_4_ administration. At 24 h after CCl_4_ treatment, cell samples were collected. (**A**) The cell viability was evaluated by an MTT assay. (**B**) The cytotoxicity was determined by an LDH assay. GSH (**C**), SOD (**D**), and MDA (**E**) contents in mice livers were measured. Data in each group are shown as means ± SD (*n* = 8 wells/group). Differences among multiple groups were analyzed with ANOVA followed by Tukey’s post hoc analysis. If there is at least one identical letter in the symbols of compared groups, there is no statistically significant difference between these groups. If there is no common letter in the compared groups, then the means between these groups are statistically different.

Consistent with the in vivo study, SA treatment statistically attenuated CCl_4_ stimulation-mediated hepatocyte injury (Figure 4A,B). Parallelly, SA-treated hepatocytes revealed increased levels of GSH and SOD with a concurrent reduction in MDA level (Figure 4C–E).

Taken together, the data suggest that SA could protect hepatocytes from oxidative stress damage induced by CCl_4_.

### 3.4. SA Could Not Directly Affect HSC Activation

**Figure 5:**
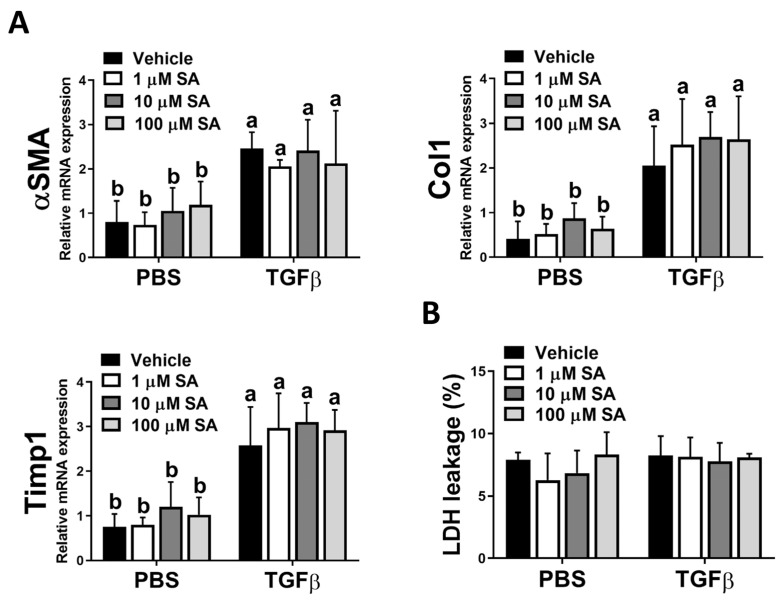
Sappanone A (SA) treatment has no significant impact on HSC activation. To activate LX-2 cells, 10 ng/mL human recombinant TGFβ was applied. Activated LX-2 cells were then treated with SA (1, 10, and 100 μM) or vehicle for 24 h. (**A**) The mRNA expression levels of αSMA, Col1, and Timp1 in LX-2 cells were measured by qRT-PCR. (**B**) The cytotoxicity was determined by an LDH assay. Data in each group are presented as means ± SD (*n* = 8 wells/group). Differences among multiple groups were analyzed with ANOVA followed by Tukey’s post hoc analysis. If there is at least one identical letter in the symbols of compared groups, there is no statistically significant difference between these groups. If there is no common letter in the compared groups, then the means between these groups are statistically different.

Since the activation of HSCs is a pivotal stage in the development of liver fibrosis, and our in vivo results showed that SA treatment reduces ECM accumulation in fibrotic livers, we next evaluated whether SA directly affects the activation of HSC. Unexpectedly, SA treatment could not affect the expression levels of fibrotic-related genes, including αSMA, Col1, and Timp1 in TGFβ-treated HSCs (Figure 5A). Furthermore, SA could not induce cell death of inactivated or activated HSCs (Figure 5B). Thus, our data indicated that SA could not directly affect HSC activation.

### 3.5. SA Decreases Hepatic Inflammatory Responses in Fibrotic Livers

**Figure 6:**
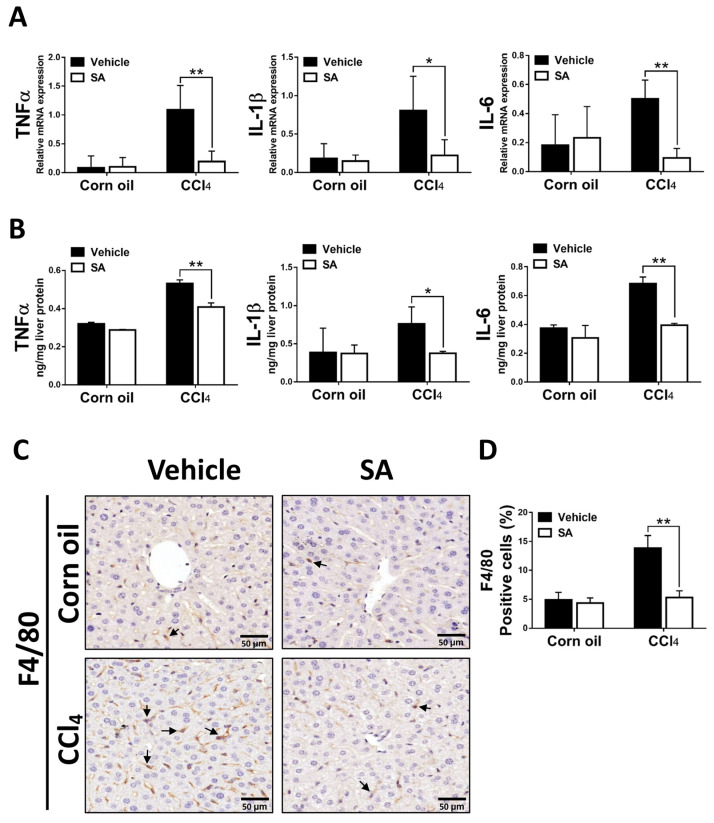
Sappanone A (SA) treatment diminishes hepatic inflammatory responses in vivo. The mRNA expression (**A**) and protein levels (**B**) of TNFα, IL-1β, and IL-6 in mice liver were measured by using qRT-PCR and ELISA. (**C**) To observe macrophage infiltration in livers of mice, the F4/80 positive macrophages in liver slides were stained with IHC. (**D**) The F4/80 positive areas were quantified. Data in each group are presented as means ± SD. * *p* < 0.05 and ** *p* < 0.01. Corn oil-treated groups (*n* = 8); CCl_4_-treated group (*n* = 10). Original magnification: ×400. The arrows in the picture show the positive-cells.

Liver fibrosis is generally preceded by chronic inflammation, and continuous inflammatory responses drive the progression of liver fibrosis [33]. SA-provided effects on CCl_4_-evoked hepatic inflammation were evaluated as the expression of key pro-inflammatory cytokines and macrophage infiltration. As we expected, animals from the SA-treated group exhibited significantly decreased mRNA and protein levels of TNFα, as well as IL-1β and IL-6 (Figure 6A,B). In line with these results, treatment with SA reduced the infiltration of F4/80 positive macrophages in fibrotic livers. Altogether, the data suggest that SA treatment decreases the pro-inflammatory environment in the liver.

### 3.6. SA Could Inhibit Macrophage Activation and Promote Polarization of Macrophages towards the M2 Phenotype by Modulating PPARγ

**Figure 7:**
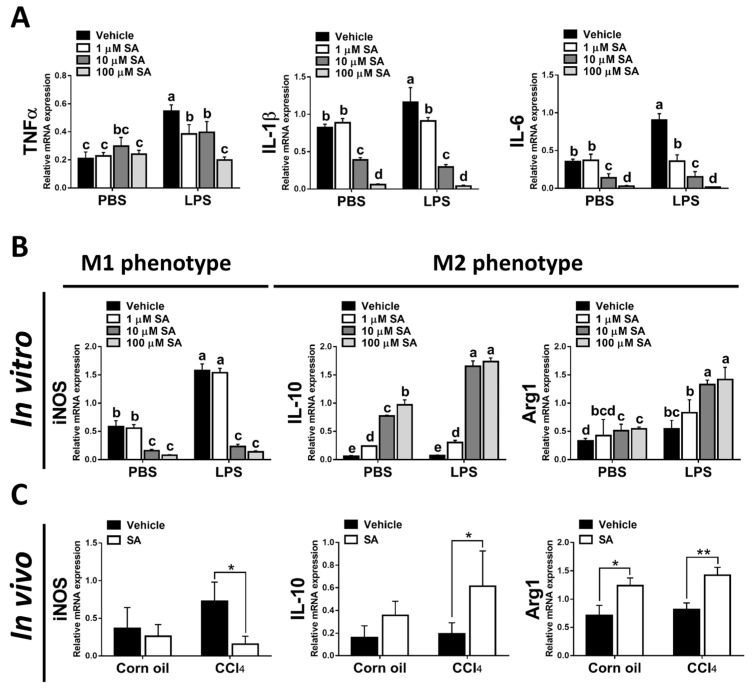

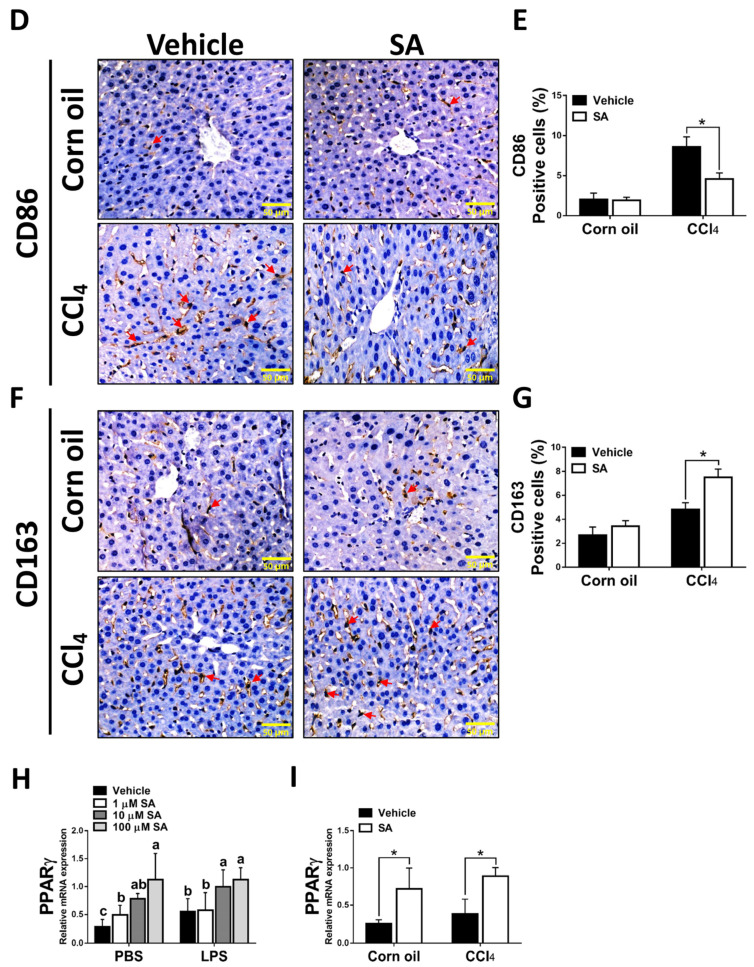
Sappanone A (SA) treatment could relieve inflammatory responses by PPARγ-mediated macrophage polarization. To demonstrate whether SA exerts anti-inflammatory properties, the RAW 264.7 cell line was treated with SA (1, 10, and 100 μM) at 30 min after LPS (1 μg/mL) stimulation (*n* = 8 wells/group). At 24 h after LPS treatment, cell samples and supernatants were collected. (**A**) The mRNA expression of TNFα, IL-1β, and IL-6 in cells was measured by qRT-PCR. The mRNA expression of iNOS (M1 phenotype maker) or IL-10 and Arg1 (M2 phenotype makers) in cells (**B**) and livers of mice (**C**) was evaluated by qRT-PCR. The CD86-positive macrophages (**D**,**E**) and CD163-positive macrophages (**F**,**G**) in liver slides were stained with IHC staining. PPARγ mRNA level was measured in cells (**H**) and livers of mice (**I**) by qRT-PCR. Data in each group are presented as means ± SD. (**A**,**B**,**H**) Differences among multiple groups were analyzed with ANOVA followed by Tukey’s post hoc analysis. If there is at least one identical letter in the symbols of compared groups, there is no statistically significant difference between these groups. If there is no common letter in the compared groups, then the means between these groups are statistically different. (**C**,**E**,**G**,**I**) Differences between the two groups were compared by two-tailed Student’s *t*-test, * *p* < 0.05 and ** *p* < 0.01. Corn oil-treated groups (*n* = 8); CCl_4_-treated group (*n* = 10). Original magnification: ×400. The arrows in the picture show the positive-cells.

It is well known that hepatic macrophages are activated in response to hepatocyte death, and M1 macrophage polarization plays a key role in the progression of liver fibrosis [34]. Therefore, we evaluated whether SA affects the activation of macrophages. Similar to in vivo results, treatment with SA markedly downregulated the mRNA expression of TNFα, IL-1β, and IL-6 in macrophages with or without LPS stimulation (Figure 7A), and the concentrations of SA used did not affect the cell viability (Supplementary Figure S3). It is well documented that macrophages stimulated with LPS undergo M1 polarization [35]. Consistently, LPS stimulation robustly increased the expression of nitric oxide synthase (iNOS, an M1 polarization marker) but diminished the mRNA expression of Interleukin 10 (IL-10) and arginase 1 (Arg1) (M2 polarization markers) in macrophages. After SA administration, reduced expression of iNOS and elevated expression of IL-10 were observed in macrophages with or without LPS stimulation, while increased expression of Arg1 was only found in LPS-treated macrophages (Figure 7B). Consistent with in vitro results, treatment with SA inhibited the M1 polarization marker and enhanced the M2 polarization markers in fibrotic livers (Figure 7C). Additionally, treatment with SA reduced the infiltration of CD86-positive macrophages (M1 macrophage polarization marker) and increased the infiltration of CD163-positive macrophages (M2 macrophage polarization marker) in fibrotic livers (Figure 7D–G).

Various transcription factors could control macrophage polarization. Accumulating studies have demonstrated that PPARγ, predominantly expressed in macrophages, is a marked feature of M2 macrophages [9]. Here, we found that SA treatment markedly up-regulated the expression of PPARγ in macrophages regardless of LPS stimulation (Figure 7H). A similar finding was found in the experiment in vivo; SA statistically enhanced the expression level of PPARγ in normal and fibrotic livers of mice (Figure 7I).

**Figure 8:**
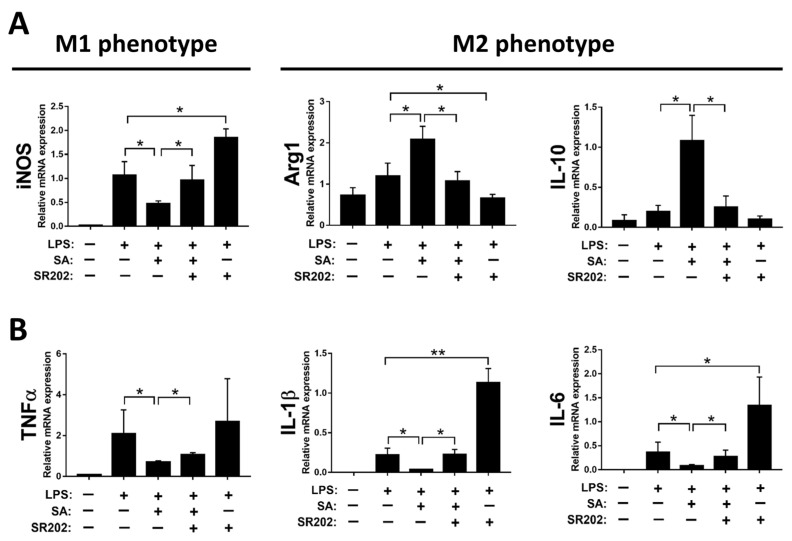
Modulation of PPARγ by sappanone A (SA) treatment affects macrophage polarization in RAW 264.7 cells. The RAW 264.7 cells were treated with 2 μM SR202 and/or 100 μM SA at 30 min and 1 h after LPS (1 μg/mL) treatment. SR202 was used to inhibit the activity of PPARγ. At 24 h after LPS stimulation, cells and supernatants were collected. (**A**) The mRNA level of iNOS (M1 phenotype maker) or IL-10 and Arg1 (M2 phenotype makers) in cells was evaluated by qRT-PCR. (**B**) The mRNA expression of TNFα, IL-1β, and IL-6 in cells was measured by qRT-PCR. Data in each group are expressed as means ± SD (*n* = 8 wells/group). * *p* < 0.05 and ** *p* < 0.01.

To confirm the PPARγ modulation by SA resulting in M2 polarization of macrophages, SR202, a selective PPARγ antagonist, was employed to inhibit the activity of PPARγ. Decreased expression of iNOS and increased expression of IL-10 and Arg1 by SA treatment were substantially abrogated by treatment with SR202 in macrophages, as displayed in Figure 8A. Similarly, decreased expression of pro-inflammatory cytokines by SA administration was reversed by SR202 treatment in macrophages (Figure 8B).

Taken together, our results indicated that SA inhibits macrophage activation and facilitates polarization of macrophages towards the M2 phenotype by modulating PPARγ.

### 3.7. Treatment with SA Promotes Fibrosis Regression

**Figure 9:**
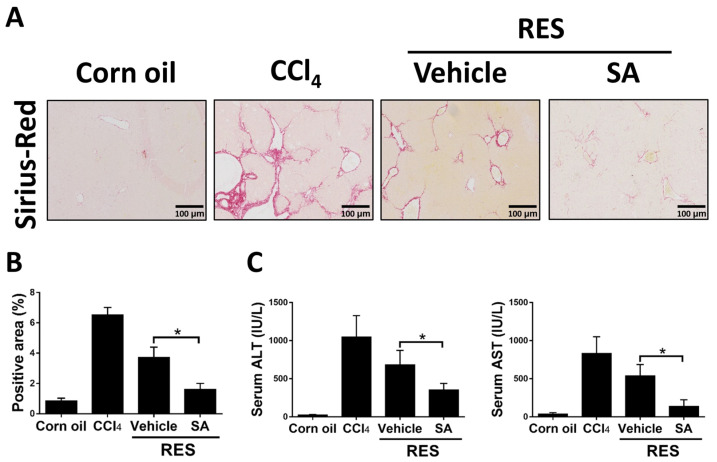
Treatment with sappanone A (SA) promotes fibrosis resolution. Mice were injected with CCl_4_ for 4 weeks, followed by discontinuation plus SA (100 mg/kg, i.p.) or vehicle every 2 days for 2 weeks (RES group). (**A**) Sirius Red-stained liver slides were used to investigate liver fibrogenesis. (**B**) Sirius Red positive areas were quantified. (**C**) The levels of ALT and AST in serum were measured. Data in each group are presented as means ± SD (*n* = 6 mice/group). * *p* < 0.05. Original magnification: ×200.

Since the above results showed that SA restrained the progression of hepatic fibrosis, we explored the effects of SA on fibrosis resolution by injecting with CCl_4_ for 4 weeks, followed by treating SA (100 mg/kg) for 2 weeks (RES group; total 6 weeks). Compared with the 4-week CCl_4_-injected group, Sirius Red staining showed that the RES group showed significantly decreased hepatic fibrosis and that such effects were further accelerated by treatment with SA (Figure 9A,B). Furthermore, treatment with SA markedly reduced serum ALT and AST levels in mice during fibrosis regression (Figure 9C). Collectively, the data indicate that SA can assist in the resolution of liver fibrosis in mice.

## 4. Discussion

In developed countries, the main causes of human death include cardiovascular disease, cancer, Alzheimer’s disease, chronic obstructive pulmonary disease, and diabetes [36,37], and 45% of these disease deaths recently are closely related to chronic fibroproliferative diseases [38]. In the liver, several chronic liver diseases are always indiscoverable without significant clinical symptoms until they progress to cirrhosis with portal hypertension and other complications, including ascites, bleeding varix, or even HCC [39,40]. During the progression of liver fibrosis, different hepatic cell types undergo impaired changes such as hepatocyte death, inflammatory cell recruitment and activation, and HSC activation [41]. Therefore, depressing or decelerating these pathological phenomena could be a potential therapeutic strategy for treating liver fibrosis. In the current study, we provided evidence that treatment with SA ameliorates CCl_4_-induced liver fibrosis, at least in part by modulating M1/M2 macrophage polarization via activating PPARγ and by inhibiting oxidative stress-induced hepatocyte cell death in mice.

Activated HSCs critically contribute to the pathogenesis of hepatic fibrosis. Stimulated by liver damage, HSCs differentiate into activated myofibroblasts and synthesize and deposit fibrillar collagen, ultimately leading to hepatic fibrosis. Therefore, restraining HSC activation has been considered a potential strategy for treating liver fibrosis. However, in the current study, data showed that treatment with SA did not directly affect the activation of HSCs (Figure 5). Of interest, inconsistent with our finding, a previous study has reported that the ethanol extract mixture of *Caesalpinia sappan* L. significantly increased fibroblast proliferation and collagen production in mouse fibroblast L929 cells [42]. Conversely, Brazilin, an extract of *Caesalpinia sappan* L., decreased collagen synthesis in a murine model of rheumatoid arthritis [43]. These studies and our findings suggest that several extracts from *Caesalpinia sappan* L. could regulate the activation of HSCs differently. Our data showed that SA reduced oxidative stress-mediated hepatocyte death and hepatic inflammation in CCl_4_-treated mice. Therefore, we suspected that SA treatment alleviated CCl_4_-induced fibrotic liver in mice through the regulation of macrophages and suppression of hepatocyte death.

During the development of liver fibrosis, macrophages are promptly activated in response to hepatocyte injury by recognizing dying cell-released damage-associated molecular patterns, subsequently secrete several inflammatory mediators, and finally recruit other immune cells into the damaged areas in the liver [44]. Hepatic macrophages with high functional plasticity have multiple roles in maintaining liver homeostasis, cytokine production, and tissue remodeling [45]. It is commonly accepted that resident hepatic macrophages are heterogeneous. Macrophages can undergo classically activated M1 polarization (pro-inflammatory) or alternatively activated M2 polarization (anti-inflammatory) in response to microenvironment cues. Recent studies suggest that macrophages switched to the M1 phenotype exert pro-inflammatory as well as pro-fibrogenic roles in the pathogenesis of liver fibrosis [46,47,48], while macrophages polarized to M2 phenotype responding to the stimulation of IL-4 or IL-13 attenuate the progression of liver fibrosis [49,50]. Hence, M1/M2 macrophage polarization provides an insight into the mechanisms regulating the resolution or progression of liver fibrosis. In general, M2 macrophage polarization is regulated by PPARγ, interferon-regulatory factor (IRF) 4, and signal transducer and activator of (IRF) 5 and transcription (STAT) 6, while STAT1 mediates M1 macrophage activation (Martinez and Gordon, 2014). PPARγ, mainly expressed by hepatic macrophages, interferes with liver fibrosis progression by regulating macrophage polarization [51]. Additionally, activated PPARγ inhibits liver fibrosis-related inflammatory responses by increasing M2 macrophage polarization [52,53]. In parallel, our results show that treatment with SA increased PPARγ activation in macrophages but not in hepatocytes and HSCs and enhanced M2 macrophage polarization that was substantially abolished by SR202 treatment. Therefore, SA-mediated PPARγ activation and M2 macrophage polarization could assist in the amelioration of CCl_4_-induced hepatic fibrosis.

In addition to macrophages, hepatocyte death could directly and indirectly activate HSCs. These cellular communications promote extracellular matrix component accumulation and induce hepatic fibrosis. In this study, the result showed that SA treatment reduced oxidative stress-induced hepatocyte cell death in CCl_4_-treated AML12 hepatocytes. This result suggests that SA ameliorates CCl_4_-induced liver fibrosis at least by inhibiting oxidative stress-induced hepatocyte cell death in mice. However, the mechanism of SA-provided protection in hepatocytes needs to be further explored in future studies. The current study also has other limitations. Firstly, CCl_4_ and SA were given intraperitoneally. Although the two chemicals were not injected on the same day, we are still not sure if the observed hepatoprotective effects of SA come from the pharmacological effects of SA itself or if the chemical reaction between CCl_4_ and SA diluted the liver damage caused by CCl_4_. Furthermore, although the hematological toxicity observed by peripheral cell counting is not competent in figuring out the alterations in quality and quantity of precursor cells in the bone marrow and reflecting toxicity to more mature hemopoietic tissue [54], it is important to investigate the short-term and long-term toxic effects of SA on the hematopoietic system, especially on erythropoiesis and leukopoiesis, complete blood counting, and renal function. Because the number of blood cells is indispensable to the complete functioning of the immune system, it facilitates deciding the end-point toxicity. However, we did not collect such data in the mice experiment. Thus, further study should solve these issues.

## 5. Conclusions

This study demonstrated that treated SA alleviates CCl_4_-induced hepatic fibrosis in mice. This result could be ascribed to the activation of PPARγ and the subsequent modulation of M1/M2 polarization. Inhibition of M1 macrophages blocks the hepatic inflammatory response, thereby reducing the activation of HSCs into myofibroblasts and diminishing collagen deposition. Also, suppression of oxidative stress-mediated hepatocyte death mediates SA-provided protective effects. Therefore, SA may be a novel therapeutic candidate for combating liver fibrosis.

## Figures and Tables

**Table 1:** Animal treatments.

Groups	Volume of Corn Oil (Body Weight)	Volume of CCl_4_ (CCl_4_:Corn Oil = 2:5, *v*/*v*) (Body Weight)	Dose of SA or Vehicle (Body Weight)	Animal Number
1	2 mL/kg, i.p.	_	PBS (200 μL, i.p.)	*n* = 8
2	2 mL/kg, i.p.	_	25 mg/kg SA (200 μL, i.p.)	*n* = 8
3	2 mL/kg, i.p.	_	50 mg/kg SA (200 μL, i.p.)	*n* = 8
4	2mL/kg, i.p.	_	100 mg/kg SA (200 μL, i.p.)	*n* = 8
5	_	2 mL/kg, i.p.	PBS (200 μL, i.p.)	*n* = 10
6	_	2 mL/kg, i.p.	25 mg/kg SA (200 μL, i.p.)	*n* = 10
7	_	2 mL/kg, i.p.	50 mg/kg SA (200 μL, i.p.)	*n* = 10
8	_	2 mL/kg, i.p.	100 mg/kg SA (200 μL, i.p.)	*n* = 10

**Table 2:** The primer sequence of real-time PCR.

Gene	Gene Accession Number	Forward (5′-3′)	Reverse (5′-3′)
*αSMA*	NM_007392	5′-TGCAGGTGATGCTGACAGAGG-3′	5′-GGGATGAGCTAGTGCTGATCTGG-3′
*Col1*	NM_007742	5′-CAGCCAATCAGCGTTCGGTA-3′	5′-CTTCATGGCGTAGTTGAATGATGTC-3′
*TGFβ*	NM_001013025	5′-AGTTGGAGCAGCTGTATCAGTGG-3′	5′-TTTAGCAAAGGCAGTCAAATCTGG-3′
*Timp1*	NM_001044384	5′-AATGACTGTTGCCAGGTGGATG-3′	5′-GGTTGCACTTCCAAATGAGGCTA-3′
*Bax*	NM_007527	5′-AAGCGCTTCGGGCCAG-3′	5′-TAGCCATGCAGGACCACGA-3′
*Bcl2*	NM_177410	5′-GGTCAGAAAGCCGTGGTTG-3′	5′-GACATGGCCTAACGTGCAG-3′
*TNFα*	NM_013693.3	5′-CGCCTCTCCTTGCTGTCACA-3′	5′-CTTTGCCTTCTGCCTCAAGT-3′
*IL-1β*	NM_008361.4	5′-GTCTACTCCCAGGTTTCTCTTCAAGG-3′	5′-GCAAATCGGCTGACGGTGTG-3′
*IL-6*	NM_001314054.1	5′-CTCGCAGCAGCACATCAACA-3′	5′-CCACGGGAAAGACACAGGTA-3′
*iNOS*	NM_010927	5′-TGCACCCAAACCGAAGTC-3′	5′-GTCAGAAGCCAGCGTTCACC-3′
*Arg1*	NM_007482	5′-GCCAAGGGTTGACTTCAAGAACA-3′	5′-AGGCTCCTCCTTTCCAGGTCA-3′
*IL-10*	NM_010548	5′-AGCAGCAGGTGTCCCAAAGA-3′	5′-GTGCTGAAGACCTTAGGGCAGA-3′
*PPARγ*	NM_133249	5′-ACGGCAAATTCAACGGCACAG-3′	5′-GAAGACTCCACGACATACTCAGCAC-3′
*hαSMA*	NM_001613	5′-ATAGAACATGGCATCATCACCAAC-3′	5′-GGGCAACACGAAGCTCATTGTA-3′
*hCol1*	NM_000088	5′-GCTTGGTCCACTTGCTTGAAGA-3′	5′-GAGCATTGCCTTTGATTGCTG-3′
*GAPDH*	NM_001411840.1	5′-ACGGCAAATTCAACGGCACAG-3′	5′-AGACTCCACGACATACTCAGCAC-3′
*hGAPDH*	NM_001256799	5′-GCACCGTCAAGGCTGAGAAC-3′	5′-TGGTGAAGACGCCAGTGGA-3′

αSMA, alpha smooth muscle actin; Col1, alpha-1 type I collagen; TGFβ, transforming growth factor β; Timp1, tissue inhibitors of metalloproteinase-1; Bax, Bcl-2-associated X-protein; Bcl2, B-cell CLL/lymphoma 2; TNFα, tumor necrosis factor; IL-1β, interleukin-1beita; IL-6, interleukin-6; iNOS, inducible nitric oxide synthase; Arg1, arginase; IL-10, interleukin-10; PPARγ, peroxisome proliferator-activated receptor gamma; hαSMA, human alpha smooth muscle actin; hCol1, human alpha-1 type I collagen; GAPDH, glyceraldehyde-3-phosphate dehydrogenase; and hGAPDH, human glyceraldehyde-3-phosphate dehydrogenase.

## Data Availability

All data are included in this manuscript.

## Supplementary Materials

The following supporting information can be downloaded at https://www.mdpi.com/article/10.3390/antiox12091718/s1. Figure S1. The daily intake of food and water on the initial day and final day; Figure S2. The liver damage was assessed by H&E staining. Original magnification: ×200; Figure S3. The cell viability was examined by an MTT assay.
