# Correction: Qi et al. Sappanone A Alleviates the Severity of Carbon Tetrachloride-Induced Liver Fibrosis in Mice. *Antioxidants* 2023, *12*, 1718

**DOI:** 10.3390/antiox14020196

**Published:** 2025-02-10

**Authors:** Jing Qi, Lanqian Li, Xueqing Yan, Wenxi Hua, Zixiong Zhou

**Affiliations:** 1 Department of Biochemistry and Molecular Biology, The School of Basic Medical Sciences, Fujian Medical University, No. 1, Xuefu North Road, University Town, Fuzhou 350122, China; yanxueqing@fjmu.edu.cn; 2 Department of Pathology and Institute of Oncology, The School of Basic Medical Sciences, Fujian Medical University, Fuzhou 350122, China; llq1998@fjmu.edu.cn (L.L.); jchwx@fjmu.edu.cn (W.H.); 3 Diagnostic Pathology Center, Fujian Medical University, Fuzhou 350122, China

In the original publication [1], there was a mistake in Figure 3D as published. The Corn oil- and SA-treated group in Figure 3D was erroneously copied from the picture of Figure 3A in another paper [2] published by the authors’ team. The reason for the misuse of the picture is that the authors’ team was processing this article [1] and another manuscript mentioned above [2] at the same time, and they accidentally reused a picture from [2]. The corrected Figure 3D appears below. The authors state that the scientific conclusions are unaffected. This correction was approved by the Academic Editor. The original publication has also been updated. 

## Figures and Tables

**Figure 3:**
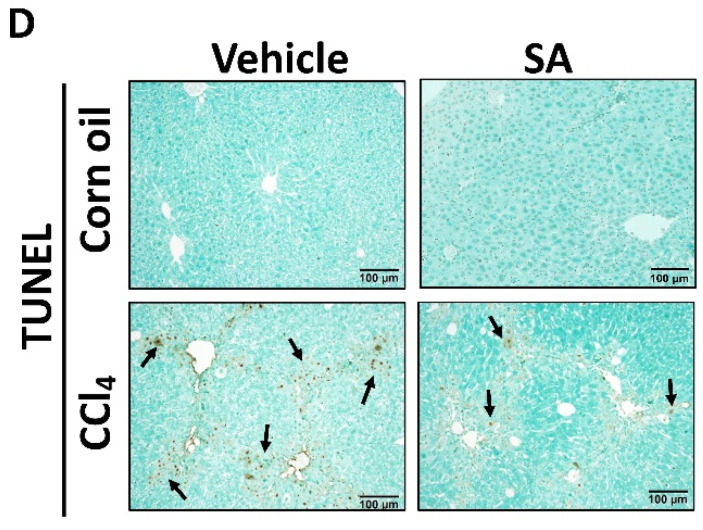
(**D**) TUNEL assay was used to explore cell death in the livers of mice.
