# Supplementary figures and images for: Sappanone A Alleviates the Severity of Carbon Tetrachloride-Induced Liver Fibrosis in Mice
Source: Antioxidants (Basel). 2023 Sep 4;12(9):1718. doi: 10.3390/antiox12091718 (PMC10526100; doi:10.3390/antiox12091718)

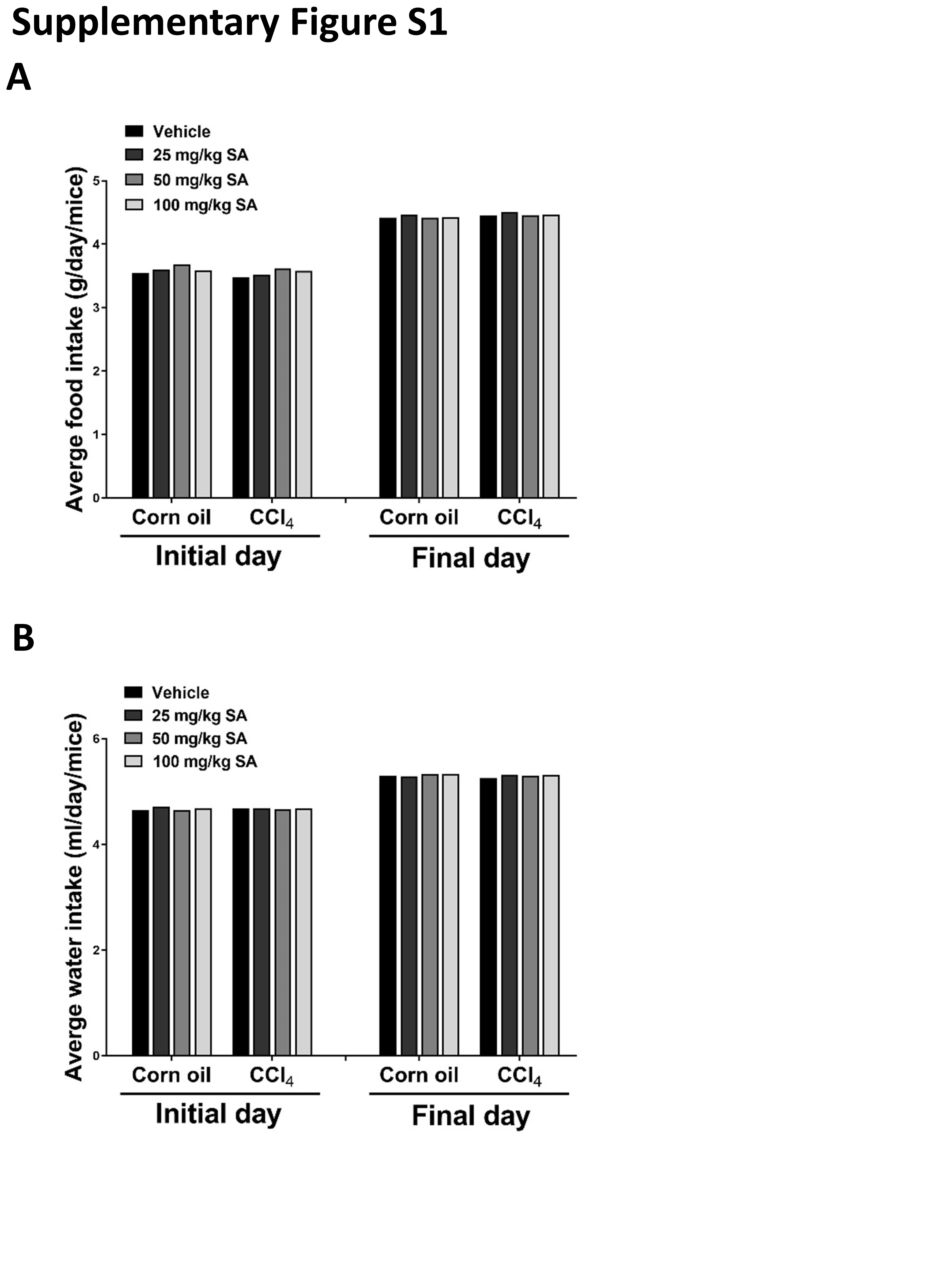

Supplement: Supplementary file 1 [file antioxidants-12-01718-s001.zip › Supplementary Figure S1.JPG]

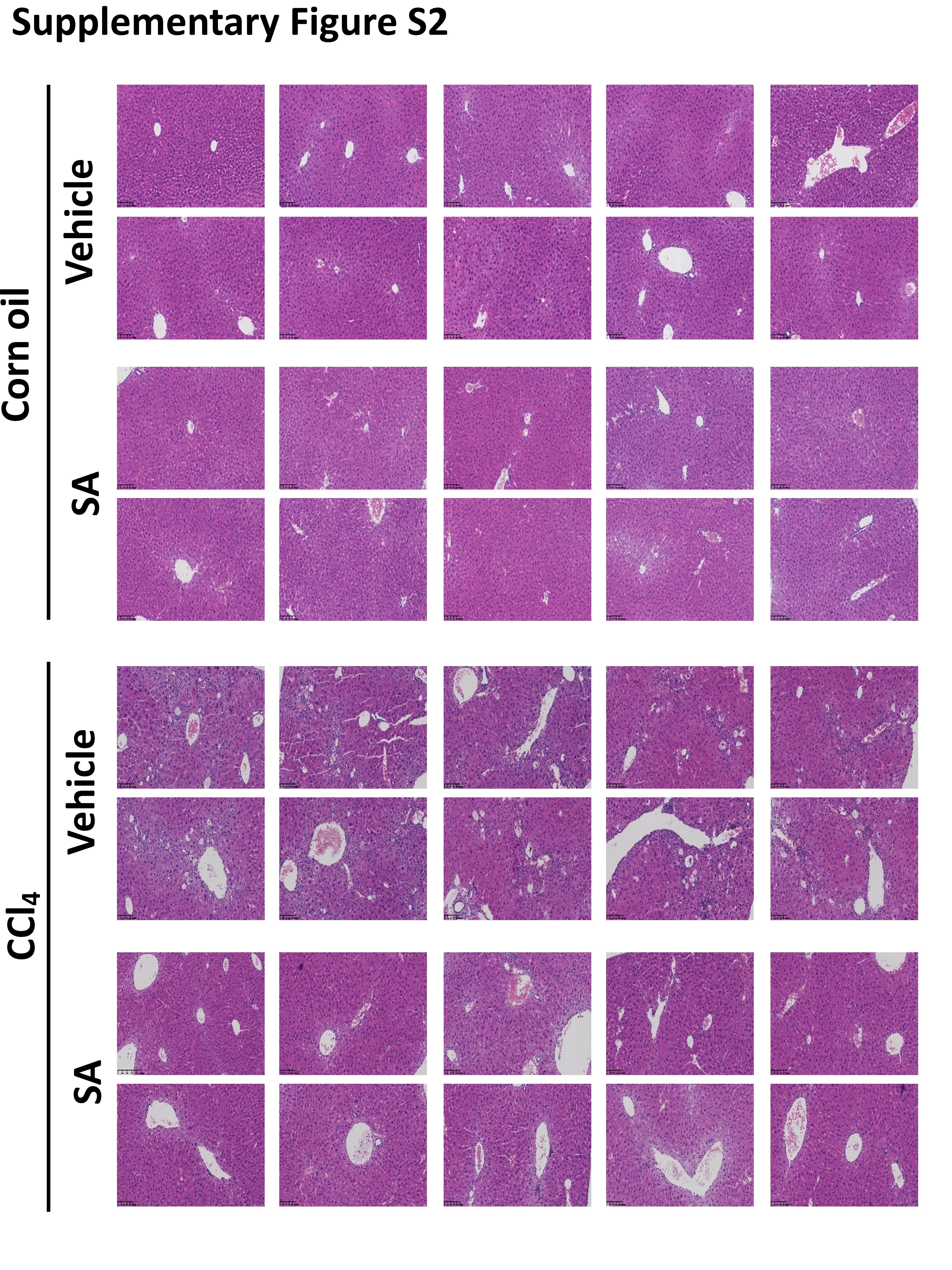

Supplement: Supplementary file 1 [file antioxidants-12-01718-s001.zip › Supplementary Figure S2.JPG]

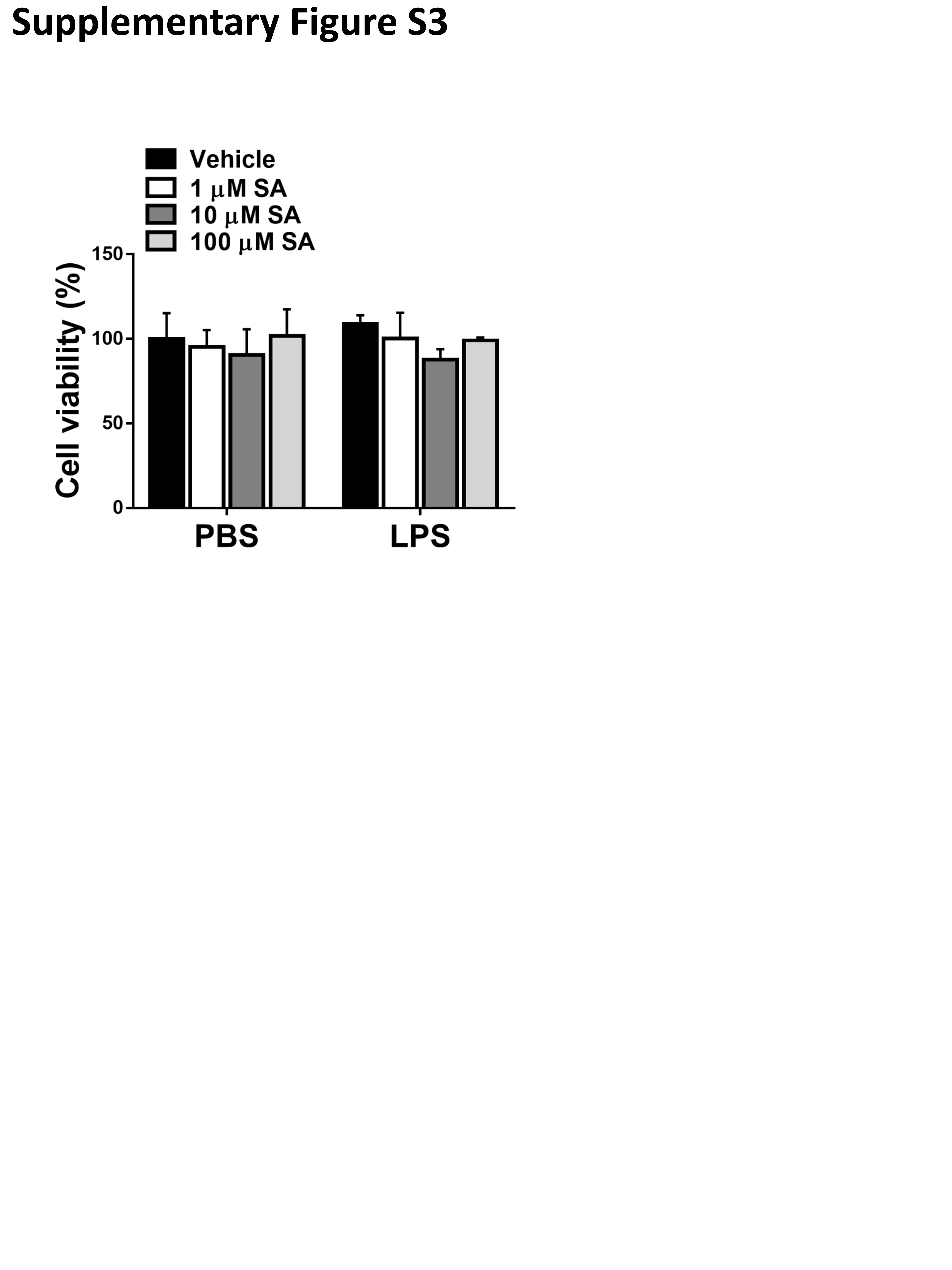

Supplement: Supplementary file 1 [file antioxidants-12-01718-s001.zip › Supplementary Figure S3.JPG]
